# Ecological and human health risk assessment of heavy metals in sewage sludge produced in Silesian Voivodeship, Poland: a case study

**DOI:** 10.1007/s10661-023-11987-z

**Published:** 2023-10-26

**Authors:** Malwina Tytła, Kamila Widziewicz-Rzońca

**Affiliations:** Institute of Environmental Engineering, Polish Academy of Sciences, 41-819 Zabrze, Poland

**Keywords:** Municipal wastewater treatment plant, Sewage sludge, Heavy metals, Ecological risk, Human health risk, Poland

## Abstract

This study aimed to assess the potential risks posed by heavy metals in sewage sludge (SS) produced by municipal wastewater treatment plants (WWTPs) in the most industrialized region in Poland, the Silesian Voivodeship. The ecological risk was assessed using three indices: the Geoaccumulation Index (*I*_*geo*_), Potential Ecological Risk Factor (ER), and Risk Assessment Code (RAC), while the health risk was estimated by using carcinogenic and non-carcinogenic risk indices. The average concentrations of metals in the sludge samples were determined revealing that Zn was the predominant element, whereas Cd and Hg were present in the lowest concentrations. The study showed that the processes used in wastewater treatment plants influenced the overall metal content and chemical speciation. According to *I*_*geo*_ values, the dewatered sludge samples exhibited higher contamination levels of Cd and Zn, while Cu and Pb were upon to a lesser extent. The ER values suggest that Cd and Hg present the highest ecological risk. Considering the chemical forms and RAC values, Ni (26.8–37.2%) and Zn (19.8–27.0%) were identified to cause the most significant risks. The non-carcinogenic risk for adults and children was below acceptable levels. However, the carcinogenic risk associated with Ni (WWTP1) for both demographic groups and Cr and Cd (WWTP2), specifically for children, exceeded the acceptable threshold. Ingestion was the primary route of exposure. Although the dewatered SS met the standards for agricultural use, there is still a risk of secondary pollution to the environment and possible adverse health effects.

## Introduction

Silesian Voivodeship is one of the sixteen provinces in Poland (Central Europe), located in the southern part of the country. This region is one of the smallest voivodeships but, due to its high industrialization, population density, and urbanization, belongs to the areas with the greatest anthropopressure (Statistics Poland, LDB, 2022). This region hosts the highest concentration of industries in the country, including metallurgy, hard coal mining, electric power, coking plants, building materials transportation, and chemical and metal industries (Baran et al., 2016). It can therefore be stated that Silesian Voivodeship constitutes the most important industrial region in Poland and also in Europe, the so-called hotspot (Tytła et al., 2016). According to the data of the Eurostat Statistics Database (Eurostat, 2021), Poland stands out with one of the highest production of municipal sewage sludge (SS) in Europe. This waste is produced as a by-product of wastewater treatment in municipal wastewater treatment plants (WWTPs) (Espinoza-Guillen et al., 2022). The largest amount of municipal SS (in dry substance (d.s.)) in the last few years is generated in Germany and France, i.e., 1 749.86 thousand tonnes d.s. (Eurostat, 2019) and 1 092.90 thousand tonnes d.s. (Eurostat, 2020), respectively.

In 2022, the number of municipal WWTPs in Poland amounted to 3,260, of which 201 are in the Silesian Voivodeship. The total mass of municipal SS generated per year amounted to 580.659 thousand tonnes d.s. of which 63.296 thousand tonnes d.s. were produced in Silesian Voivodeship. Moreover, the number of industrial WWTPs in the country is 854 of which 124 are located in the Silesian region (Statistics Poland, LDB, 2022). Therefore, particular emphasis should be placed on wastewater generated by various industrial sectors. This is due to the fact that these effluents substantially impact the composition of raw wastewater discharged into municipal WWTPs. This directly affects the chemical characteristics of the resulting sludge (Tytła et al., 2019).

From the economic and environmental point of view, the most attractive method for sewage sludge disposal is its land application, as a fertilizer or conditioner. This way of sludge management is not as expensive as others and characterized by high efficiency (Geng et al., 2020; Xuan et al., 2023). Moreover, it also seems very beneficial, especially for developing countries (Yakamercan et al., 2021). However, one of the great concerns is the presence of heavy metals (HMs) (Espinoza-Guillen et al., 2022). The main sources of these elements in sewage sludge are industrial and domestic wastewater and to a lesser extent the corrosion of sewage pipes and surface runoff from roads and urbanized areas (Tytła, 2019; Yakamercan et al., 2021). Taking into account the fact that 26.7% of SS produced in Polish municipal WWTPs is used for agricultural purposes, the presence of HMs from industrial effluents may pose a serious threat, both in the environmental and human health dimensions (Bochenek et al., 2022). In recent years great scientific attention has also been put on the chemical speciation forms of HMs occurrence in sludge, which gives information on their toxicity, mobility, and bioavailability (Nkinahamira et al., 2019; You et al., 2021). This fact was very often ignored since the quantitative analysis of the total HM content was rather expensive.

The only way to identify and eliminate the potential threats associated with HMs contained in municipal sewage sludge, before its land application, is to assess the ecological and human health risks. The analysis of potential ecological risk is crucial for safeguarding the water and soil environment against potential secondary pollution caused by the presence of heavy metals (Yakamercan et al., 2021). On the other hand, absorption of HMs via accidental ingestion, inhalation, or dermal contact with sewage sludge leads to their accumulation in the human fatty tissues and may negatively influence their nervous system, immune and endocrine systems, and their hematopoietic function (Zhang et al., 2020; Yakamercan & Aygün 2023). Therefore, it is highly recommended to conduct a full risk assessment.

In the scientific literature, there are several articles that focus on evaluating the potential ecological risk associated with HMs in SS used for agricultural purposes. However, there is a scarcity of research that would focus on binding these elements in sludge and its influence on the level of the final ecological risks (i.e., Wang et al., 2006; Gwebu et al., 2017; Tytła et al., 2023). There are also very few studies that specifically focus on the assessment of human health risks of HMs in sewage sludge (i.e., Kendir et al., 2015; Duan et al., 2017; Duan & Feng, 2021; Yakamercan et al., 2021; Espinoza-Guillen et al., 2022; Qayoom et al., 2022; Yakamercan & Aygün 2023); however, none of them deals with SS produced in the municipal WWTPs located in Poland. This research generates new knowledge about the potential risks associated with HMs in sewage sludge in this country.

The main objectives of this study are (a) to determine the total concentrations of Cd, Cr, Cu, Ni, Pb, Zn, and Hg in SS samples collected from two municipal wastewater treatment plants in Silesian Voivodeship (Poland) and their distribution in particular chemical speciation fractions; (b) to assess the potential ecological risk posed by analyzed HMs; and (c) to estimate the non-carcinogenic and carcinogenic human health risks from HMs contaminants in the sludge used for agricultural purposes, via accidental ingestion, inhalation, or dermal contact exposure.

## Materials and methods

### Study area and sampling

**Fig. 1:**
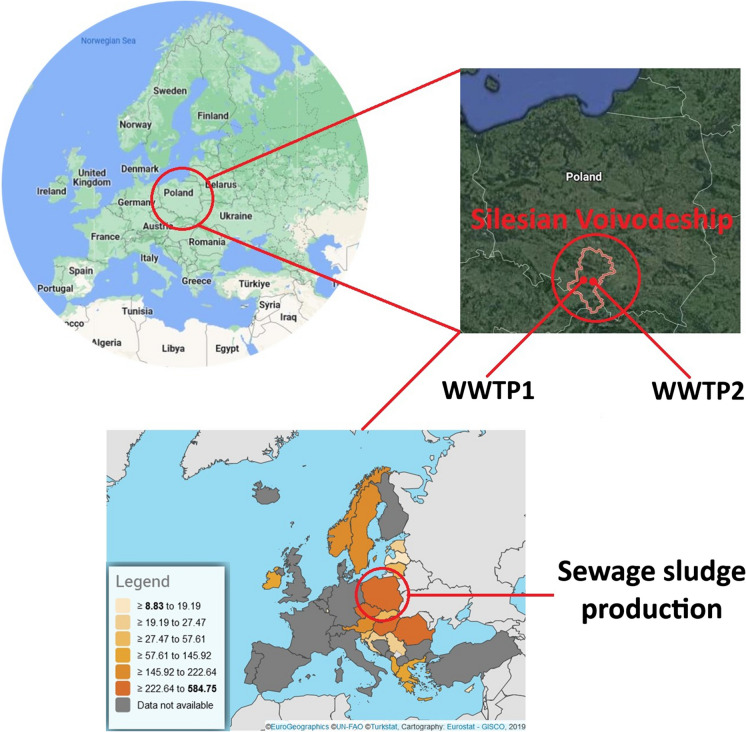
The location of the study area. The source file of the maps placed on the top is Google Maps, (n.d.) (https://www.google.pl/maps/), while the map placed on the bottom is Eurostat (https://ec.europa.eu/eurostat/)

The study area for this research is the Silesian Voivodeship, located in the southwestern part of Poland. Fig. 1 illustrates the geographical location of the study area and the wastewater treatment plants under investigation. We also present the amount of sewage sludge (in thousand tonnes d.s.) produced in selected European countries, including Poland (Eurostat, 2021). The WWTPs considered in this study were located a short distance from each other, approximately 25 km. Each of the WWTPs collects wastewater which was discharged from the neighboring towns.

In the study, sludge samples were collected from two municipal WWTPs at every stage of processing throughout the technological line. The sampling was conducted in two seasons, namely, winter and summer, in 2021. The main operating parameters of the WWTPs are presented in Table 1. The distance between the WWTPs is small, so the characteristics of their catchment areas are similar. During the field research, seven sludge samples were gathered (every time), i.e., the primary sludge (S1), thickened primary sludge (S2), excess sludge (S3), thickened excess sludge (S4), mixed sludge (thickened primary and thickened excess sludge) (S5), anaerobically digested (stabilized) sludge (S6), and the dewatered sludge (S7). Each sample consisted of several subsamples collected at regular intervals. The collected SS samples were carefully placed in pre-cleaned polypropylene and then stored in a portable refrigerator at a temperature of 4 °C. After completion of the field research, collected samples were transported to a laboratory for further analysis.

**Table 1:** The crucial operational parameters of municipal wastewater treatment plants

Parameter	Unit	WWTP1	WWTP2
Population equivalent (P.E.)	-	156,627	218,950
Wastewater treatment method	-	Activated sludge	Activated sludge
Average daily flow	m^3^·day^-1^	35,601	31,139
Share of industrial wastewater	%	10	7
Stabilization time	day	25–26	40–42
Stabilization temperature	°C	36	37

Data obtained from the WWTPs

### Sewage sludge analysis

In this study, several physicochemical parameters were analyzed in the collected sewage sludge samples. To measure pH and Eh, a digital CPR-411 meter (Elmetron, Poland) equipped with IJ-44A and ERS-2 electrodes (Elmetron, Poland) was used. The potentiometric method was used for these measurements. The determination of DM (dry matte) and OM (organic matter) content followed the Polish standards: PN-EN 12880:2004 (2004a) and PN-EN 12879:2004 (2004b), respectively. For the determination of TOC (total organic carbon) content, the standard PN-EN 13137:2004 (2004c) was applied, using the TOC analyzer 5000A (Shimadzu, Japan).

These analytical methods and instruments were utilized to assess the specified physicochemical parameters, enabling a comprehensive understanding of the characteristics and composition of the sewage sludge samples in this study.

### Determination of heavy metals

Heavy metals for chemical analysis were selected based on the requirements for sewage sludge intended for agricultural purposes and/or for reclamation, which have been defined in the national (Regulation of the Minister of Environment of 6th February 2015 on the Municipal Sewage Sludge) and the European Union (EU) (Council Directive of 12th June 1986) acts. The overall concentrations of heavy metals in different SS samples were determined according to the following steps. In the first place, the sludge sample was dried in a laboratory dryer SUP-100G at 105 °C (Wamed, Poland), then ground in a mortar grinder Pulverisette 2 (Fritsch, Germany), and sieved through sieves with a diameter of 0.2 mm. In the next step of the analysis, a 0.2-g sludge sample was subjected to microwave-assisted digestion, also known as mineralization. The digestion process was carried out using 15-mL hydrochloric acid (HCl) with a concentration of 35–38% and 5-mL nitric acid (HNO_3_) with a concentration of 65%. The mineralization process was performed in the Multiwave 3000 digestion system by Anton Paar GmbH (Austria). In the third step, the total concentrations of the HMs in the obtained solution were determined. The analysis was performed using two different techniques: inductively coupled plasma atomic emission spectroscopy (ICP-OES) with the Avio 200 system by PerkinElmer, Inc. (USA) and cold vapor atomic absorption spectrometry (CVAAS). The detection limits (LODs) for the studied HMs were as follows: 0.004 mg L^−1^ for Cd, 0.006 mg L^−1^ for Cr, 0.005 mg L^−1^ for Cu, 0.007 mg L^−1^ for Ni, 0.009 mg L^−1^ for Pb, 0.008 mg L^−1^ for Zn, and 0.0001 mg L^−1^ for Hg. These values indicate the lowest concentration of each element that can be reliably detected and quantified by the analytical instruments used in the study. The recovery rates (R) for analyzed HMs in ERM-CC144, a certified reference material (CRM) by the Joint Research Center (JRC, Belgium), were in the range of 87 to 101%. The values of the relative standard deviation (RSD) for analyzed HMs did not exceed 3%. The operational parameters for ICP-OES were described in detail, in the previous study (Tytła et al., 2023). All chemical reagents used in this study, except reference materials, were manufactured by Avantor Performance Materials Poland S.A. (formerly POCH; Poland).

### Sequential extraction procedure

To determine the chemical speciation forms of HMs in different types of sewage sludge samples, the study utilized the ultrasound-assisted BCR sequential extraction method, which was described in the previous work (Tytła et al., 2022). The original extraction procedure was recommended by the Community Bureau of Reference, now the Standards, Measurements and Testing Programme, and was based on the method developed by Tessier et al. (1979). Moreover, this procedure is well-known and widely used for assessing the fractionation of metals in various environmental matrices (Ure et al., 1993; Álvarez et al., 2002). That was the main reason why we used this method in our study.

**Fig. 2:**
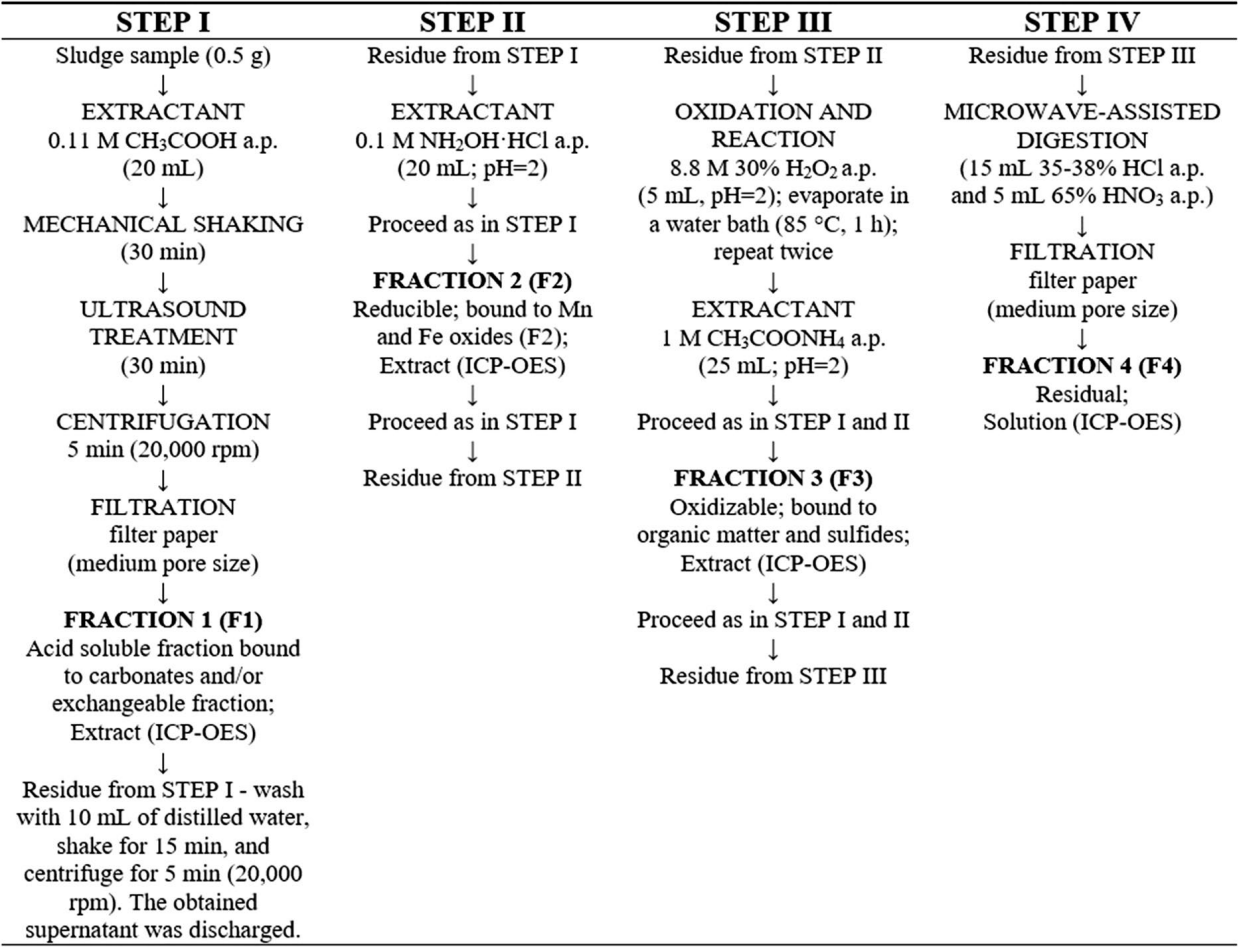
The procedure of the ultrasound-assisted BCR sequential extraction (Tessier et al., 1979; Tytła et al. 2022)

In this study, all analyses were performed in duplicate with reagent blanks. The content of HMs in the extracts was determined using a similar approach as in the case of solutions obtained from microwave digestion. The ultrasound-assisted procedure of BCR sequential extraction is shown in Fig. 2.

### Assessment of potential ecological risk

The potential ecological risk of heavy metals in sewage sludge samples was assessed using three different risk indices that were initially developed for the evaluation of ecological risk in sediments and soils.

### Geoaccumulation Index  (I_geo_)

The Geoaccumulation Index (*I*_*geo*_) was proposed by Müller (1969). This index is used as a quantitative measure to assess the degree of contamination or enrichment of metals that are present in a specific environment (Xuan et al., 2023) (Eq. 1):

1 $${I}_{geo}={\mathit{\log}}_2\left(\frac{C_n}{1.5{B}_n}\right)$$

where *C*_*n*_ is the measured concentration of individual metal in a sample; *B*_*n*_ is the concentration of individual metal in the geochemical background (earth crust) or in the reference environment (soil) (Kabata-Pendias, 2011).

*I*_*geo*_ ≤ 0 — practically uncontaminated (PUC); 0 < *I*_*geo*_ ≤ 1 — uncontaminated to moderately contaminated (U-MC); 1 < *I*_*geo*_ ≤ 2 — moderately contaminated (MC); 2 < *I*_*geo*_ ≤ 3 — moderately to heavily contaminated (M-HC); 3 < *I*_*geo*_ ≤ 4 — heavily contaminated (HC); 4 < *I*_*geo*_ ≤ 5 — heavily to extremely contaminated (H-EC); *I*_*geo*_ > 5 — extremely contaminated (EC) (Müller, 1969).

### Potential Ecological Risk Factor

The Potential Ecological Risk Factor (ER) was proposed by Håkanson (1980). This method is used to assess the ecological risk for a given metal in the various environmental matrices. ER includes both the concentration of a particular metal and its toxicity (Duan & Feng, 2022) (Eq. 2):

2 $$ER={T}_f^i CF$$

where CF — Contamination Factor — the quotient of the concentration of metal in a sample and in the reference or baseline (*B*_*n*_) (Håkanson, 1980); *T* is the toxic response factor: Cd = 30, Cr = 2, Cu = 5, Pb = 5, Zn = 1, and Hg = 40.

ER ≤ 40 — low risk (LR); 40 < ER ≤ 80 — moderate risk (MR); 80 < ER ≤ 160 — considerable risk (CR); 160 < ER ≤ 320 — high risk (HR); ER > 320 — very high risk (VHR) (Håkanson, 1980).

### Risk Assessment Code

The Risk Assessment Code (RAC) was introduced by Perin et al. (1985). This index defines the risk level based on the percentage share of individual metals in the most mobile of speciation fractions (Kowalik et al., 2021) (Eq. 3):

3 $$RAC=F1\left(\%\right)$$

where *F*1 — is the percentage concentration of metal in the fraction F1.

RAC ≤ 1% — no risk (NR); 1% < RAC ≤ 10% — low risk (LR); 10% < RAC ≤ 30% — medium risk (MR); 30% < RAC ≤ 50% — high risk (HR); RAC > 50% — very high risk (VHR) (Perin et al., 1985).

### Assessment of human health risk

The heavy metals considered in this research were described as potentially hazardous pollutants. The United States Environmental Protection Agency (USEPA) established the ceiling concentration limits for ten metal(loid)s in biosolids (sewage sludge) applied to land, i.e., to condition the soil or fertilize crops and other vegetation (As, Cd, Cr, Cu, Hg, Mo, Ni, Se, and Zn). All elements discussed in this study were listed in the “EPA Part 503 Biosolids Rule” (USEPA, 1995). Moreover, both the exposure and the non-carcinogenic and carcinogenic health risks were assessed based on the mean concentrations of HMs in the dewatered sewage sludge samples collected from two WWTPs, in two seasons.

### Exposure assessment

The main pathways through which the heavy metals contained in sewage sludge can enter the human body are accidental ingestion, inhalation, and dermal contact. Calculations of the average daily dose for the individual exposure route (*ADD*_*ing*_, *ADD*_*inh*_, and *ADD*_*dermal*_) and the average daily total exposure dose (ADD) can be performed using the equations listed below (Eqs. 4–7) (USEPA, 1989):

4 $${ADD}_{ing}=\frac{C\times {IR}_{ing}\times EF\times ED}{BW\times AT}\times CF$$

5 $${ADD}_{inh}=\frac{C\times {IR}_{inh}\times EF\times ED}{PEF\times BW\times AT}$$

6 $${ADD}_{dermal}=\frac{C\times SA\times AF\times ABS\times EF\times ED}{BW\times AT}\times CF$$

7 $$ADD={ADD}_{ing}+{ADD}_{inh}+{ADD}_{dermal}$$

where *ADD*_*ing*_, *AD*_*inh*_, and *ADD*_*dermal*_ are the average daily dose (mg kg^−1^ day^−1^) for ingestion, inhalation, and dermal contact, respectively; ADD is the average daily total exposure dose (mg kg^−1^ day^−1^). The parameters applied to estimation of the ADDs are shown in Table 2.

**Table 2:** The parameters applied to the estimation of ADDs

Parameter	Symbol	Unit	Parameter value Adults/Children	References
Concentration of HM in SS	C	mg kg^−1^	-	This study
Ingestion rate	*IR*_*ing*_	mg day^−1^	100/200	USEPA (1991) USEPA (2002)
Inhalation rate	*IR*_*inh*_	m^3^ day^−1^	20/7.6	USEPA (2002) Espinoza-Guillen et al. (2022)
Exposure frequency	EF	day year^−1^	350	USEPA (1991) USEPA (2002)
Exposure duration	ED	year	30/6	USEPA (2002)
Exposed skin area	SA	cm^2^	5700/2800	USEPA (2002)
Skin adherence factor	AF (soil)	mg cm^−2^	0.07/0.2	USEPA (2002)
Dermal absorption factor	ABS	-	0.001	USEPA (2002)
Particulate emission factor	PEF	m^3^ kg^−1^	1.36 × 10^9^	USEPA (2002)
Body weight	BW	kg	70/16	USEPA (1989)
Averaging time	AT	days	70 × 365/carcinogens ED × 365/non-carcinogens	USEPA (1989)
Conversion factor	CF	kg mg^−1^	10^−6^	USEPA (1989)

### Risk characterization

In accordance to the International Agency for Research on Cancer (IARC), among the analyzed metals: Cd, Cr, Ni, and Pb are classified as carcinogenic pollutants, where Cd, Cr (Cr^+6^), and Ni belong to group 1 while Pb to group 2A (IARC, 2012). Therefore, the carcinogenic risk was assessed only for the above-mentioned heavy metals, whereas for the rest of the analyzed elements (Cu, Zn, Hg), the non-carcinogenic risk was estimated.

### Non-carcinogenic risk assessment

To assess the non-carcinogenic risk posed by each of the exposure pathways and evaluate the overall non-carcinogenic effects of the analyzed HMs, the hazard quotient (HQ) and hazard index (HI) were calculated (Eqs. 8 and 9) (USEPA, 1989; USEPA, 2001):

8 $${HQ}_{ij}=\frac{ADD_{ij}}{RfD_{ij}}$$

9 $$HI=\sum {HQ}_{ij}$$

where *RfD* is the reference dose of individual metal (mg kg^−1^·day^−1^), to which a human can be exposed (per day), during his lifetime without harm (Table 3).

**Table 3:** The RfD and SF values of heavy metals (Yakamercan et al., 2021; Miletić et al., 2023; RAIS, 2023)

Metal	*RfD*_*ing*_	*RfD*_*inh*_	*RfD*_*dermal*_	*SF*_*ing*_	*SF*_*inh*_	*SF*_*dermal*_
	mg·kg^−1^·day^−1^			kg·day·mg^−1^		
Cd	-	-	-	6.30	6.30	-
Cr	-	-	-	5.00 × 10^-1^	42.0	20.0
Cu	4.00 × 10^-2^	4.02 × 10^-2^	1.20 × 10^-2^	-	-	-
Ni	-	-	-	1.70	8.40 × 10^-1^	42.5
Pb	-	-	-	8.50 × 10^-3^	4.20 × 10^-2^	8.50
Zn	3.00 × 10^-1^	3.00 × 10^-1^	6.00 × 10^-2^	-	-	-
Hg	3.00 × 10^-4^	3.00 × 10^-4^	1.84 × 10^-3^	-	-	-

If the HQ or HI value exceeds 1, there may be potential non-carcinogenic effects, while if the HQ or HI value is less than 1, there is no experience of any health risks for exposure to non-carcinogenic heavy metals (USEPA, 1989; USEPA, 2001).

### Carcinogenic risk assessment

To assess the carcinogenic risk posed by each of the exposure pathways and evaluate the overall carcinogenic effects of the studied HMs, the carcinogenic risk (CR) and total carcinogenic risk (TCR) were estimated (Eqs. 10 and 11) (USEPA, 1989; USEPA, 2001):

10 $${CR}_{ij}={ADD}_{ij}\times SF$$

11 $$TCR=\sum {CR}_{ij}$$

where *SF* is the cancer slope factor (mg kg^−1^·day^−1^) through ingestion, inhalation, and dermal contact of individual heavy metal (Table 3).

If the CR or TCR values are greater than 1 × 10^−4^, the carcinogenic risk occurs; if the values are between 1 × 10^−6^ and 1 × 10^−4^, the carcinogenic risk is acceptable; if the values are below 1 × 10^−6^, the risk is not carcinogenic (USEPA, 1989; USEPA, 2001).

### Statistical analysis

Risk assessments and statistical analyses were conducted using two software programs: MS Excel by Microsoft and Statistica ver. 12.0 by StatSoft, which provides tools and functions for data analysis, visualization, and statistical calculations. The statistical analyses were performed with a confidence interval of 95% and a significance level of *α* = 0.05.

## Results and discussion

### Sewage sludge characteristics

The results presented in the scientific studies have shown that the total content and chemical forms of HMs in sewage sludge are strongly influenced by various characteristics of the sludge itself. These characteristics include pH, TOC, OM, and Eh. Moreover, these properties can change at the different stages of sewage sludge processing (Wang et al., 2006; Tytła et al., 2016). This in turn can significantly influence the assessment of ecological and human health risks, particularly when the dewatered sludge is utilized for agricultural purposes. The physicochemical parameters of sludge samples are shown in Table 4. Based on the presented data, it can be stated that sludge samples from two municipal WWTPs have similar characteristics. The values of particular sludge parameters are typical and comparable to those presented in other scientific works (Gusiatin et al., 2018; You et al., 2021; Xuan et al., 2023). Moreover, the pH values of digested SS samples and temperature during anaerobic digestion were rather within the levels considered optimal for this process. The optimal pH values and temperature during anaerobic digestion are 6.8 to 7.4 and 33 to 37 °C, respectively (Cieślik et al., 2015). In this study, the only exception was the values of Eh. The redox potential of digested sludge samples is typically maintained at lower values. According to literature data, the optimal range for this parameter in anaerobic digestion is around − 520 to − 530 mV (Cieślik et al., 2015). This is essential for the activity of anaerobic microorganisms involved in the degradation of organic matter. In this case, the stabilization time was too short or there are some factors causing disturbances in the process. All these factors may have influenced the final HM concentrations. That is why, it is so important to monitor changes in HM content throughout the processing line of WWTP.

**Table 4:** Characteristics of sludge samples from two WWTPs

S	pH		Eh		DM		DOM		TOC	
	-		mV		%		%_DM_		%	
	WWTP1	WWTP2	WWTP1	WWTP2	WWTP1	WWTP2	WWTP1	WWTP2	WWTP1	WWTP2
S1	6.8	7.0	− 210	− 263	2.5	0.3	63.4	40.5	36.0	29.9
S2	6.1	6.7	− 221	− 268	3.2	3.6	71.9	73.9	34.9	39.0
S3	7.1	6.9	− 90	− 112	0.9	1.0	66.4	65.7	29.9	28.7
S4	6.9	6.8	− 103	− 111	3.6	3.1	70.3	68.9	31.5	33.5
S5	6.3	5.6	− 185	− 181	3.5	4.3	70.2	73.0	32.8	36.9
S6	6.7	7.0	− 234	− 288	2.2	2.6	60.2	58.6	27.3	27.2
S7	7.6	7.7	− 164	− 174	20.7	19.4	61.0	59.8	29.6	28.8

### Heavy metal concentrations

Based on the obtained results, there were no notable seasonal variations in the total concentrations of heavy metals in municipal sewage sludge produced at WWTPs. The only exception is concerning Zn levels at S1 during the summer season at WWTP1, and at points S3 and S4 during the winter season at WWTP2. The concentrations of this element at these points were slightly higher compared to the entire measurement period. Therefore, the mean values of HMs concentrations were used for further calculations (Table 5).

**Table 5:** Mean concentrations of heavy metals in sludge samples from two WWTPs

HMs	S1	S2	S3	S4	S5	S6	S7
	WWTP1						
	mg·kg^−1^						
Cd	3.0	2.8	3.1	3.6	3.4	3.7	4.8
Cr	51.1	107.2	58.3	65.6	59.0	77.9	78.9
Cu	185.1	172.7	197.3	216.9	208.1	234.1	240.1
Ni	94.0	94.3	100.6	113.9	88.7	126.1	136.5
Pb	48.3	61.8	48.5	58.1	54.9	63.9	71.8
Zn	1971.4	850.9	818.8	933.1	824.9	1059.0	1109.6
Hg	0.33	0.34	0.21	0.25	0.20	0.27	0.28
	WWTP2						
	mg·kg^−1^						
Cd	6.6	7.3	12.7	15.7	11.9	15.6	17.3
Cr	47.9	61.0	193.4	296.6	199.9	226.3	263.3
Cu	97.8	127.7	187.7	258.4	167.6	230.4	249.5
Ni	12.3	16.1	22.7	28.8	26.0	46.2	49.5
Pb	88.9	129.9	180.8	227.9	176.7	266.6	290.3
Zn	866.3	1096.8	1721.4	1870.9	1419.3	1978.8	2062.8
Hg	0.04	0.09	0.10	0.08	0.05	0.09	0.09

The average levels of heavy metals concentrations in the sewage sludge samples collected throughout the processing line of the studied municipal wastewater treatment plants were as follows: Zn > Cu > Ni > Cr > Pb > Cd > Hg (WWTP1) and Zn > Pb > Cu > Cr > Ni > Cd > Hg (WWTP2). The differences observed in the concentrations of HMs in sludge samples result from different characteristics of wastewater that enter a given wastewater treatment plant. However, sludges were characterized with higher concentrations of Zn (WWTP1 and WWTP2) and Cu (WWTP1) or Pb (WWTP2) while in both cases with lower for Cd and Hg. A similar tendency was noted in a previous study, where HM concentrations were as follows: Zn > Cu > Pb > Ni > Cr > Cd > Hg (Tytła, 2019). The obtained results are in line with findings reported in other scientific studies. For example, in the research by Duan and Feng (2021) and by Qayoom et al. (2022) conducted in China and India, respectively, zinc consistently appears as the element with the highest mean concentration, while the order of other HMs differs between the two studies. Also, other group of scientists, who studied SS from 10 WWTPs in China, indicated that Zn and Cu appear in the highest concentrations (Zn > Cu > Cr > Ni > Pb > As > Hg > Cd) (Li et al., 2019). However, in this research, the highest concentrations of metals were found at sampling points S6 (digested sewage sludge) and S7 (dewatered sewage sludge). In the case of digested SS, the probable cause of the increase of HMs concentrations was their adsorption on the flocs surface, whereas, for the dewatered one, the main cause was the increase of DM content, as a result of the dehydration process (Tytła et al., 2023). It was also revealed that the concentrations of HMs in the dewatered sludge (S7) from the studied WWTPs are higher than those from WWTPs, located in other regions of the country, e.g., in the Pomeranian Voivodeship (north Poland) (Milik et al., 2017), Warmian-Masurian Voivodeship (north-east Poland) (Gusiatin et al., 2018), and Świętokrzyskie Voivodeship (southern part of central Poland) (Kowalik et al., 2021). Furthermore, the results of this study indicate that the total contents of heavy metals in the dewatered sludge samples are within the acceptable range for land application in Poland (J. L. 2015, Item 257) and European Union (Directive 86/278/EEC). However, the fact that the permissible limits are not exceeded does not imply that these metals cannot pose potential threats to the environment and human health.

Table 6 shows the outcomes of the statistical analysis, specifically Pearson’s correlation analysis, performed for the studied HMs. In the case of sludge samples from WWTP1, significant positive correlations were found between Cd–Cu (0.90), Cd–Ni (0.88), Cd–Pb (0.77), Cu–Ni (0.86), and Ni–Pb (0.77). In turn, in the case of WWTP2, strong correlations were found between all of the analyzed HMs, except Ni–Cr, whereas Hg did not correlate significantly with other elements. The research findings are consistent with those presented by other scientists (Zhang et al., 2020; Yakamercan et al., 2021). The strong correlations between HMs indicate that their presence and distribution in sewage sludge are influenced by similar factors. This could be due to similar chemical properties, common sources of contamination, or similar transport mechanisms within the wastewater treatment processes (Nkinahamira et al., 2019; Espinoza-Guillen et al., 2022). To verify the above-presented assumptions, in-depth statistical analysis should be applied (principal component analysis; PCA), but this requires a larger sample size.

**Table 6:** Correlation matrix of heavy metals content in sludge samples from WWTPs

	Cd	Cr	Cu	Ni	Pb	Zn	Hg
WWTP1							
Cd	1.00						
Cr	0.05	1.00					
Cu	0.90*	− 0.11	1.00				
Ni	0.88*	0.21	0.86*	1.00			
Pb	0.77*	0.66	0.62	0.77*	1.00		
Zn	− 0.09	− 0.40	− 0.17	− 0.06	− 0.29	1.00	
Hg	− 0.15	0.52	− 0.37	0.03	0.24	0.55	1.00
WWTP2							
Cd	1.00						
Cr	0.96*	1.00					
Cu	0.98*	0.96*	1.00				
Ni	0.89*	0.75	0.83*	1.00			
Pb	0.97*	0.88*	0.94*	0.96*	1.00		
Zn	0.98*	0.92*	0.97*	0.88*	0.97*	1.00	
Hg	0.52	0.43	0.57	0.45	0.56	0.66	1.00

*Significant correlation at *p* < 0.05

However, last year in Poland, the amount of industrial wastewater discharged into the sewer system amounted to 103,180 dam^3^ of which 8,711 dam^3^ was discharged in the Silesian Voivodeship (Statistics Poland, LDB, 2022). Moreover, currently, in Poland, there are 2,040 plants of which 1,149 do not have their own WWTP and discharge industrial wastewater directly into the sewer system. Even though the Silesian region is one of the smallest voivodeships in the country, the number of such plants amounted here to 197 of which 79 discharge wastewater directly to the sewage system. This puts the Silesian region in third place in the country, in this regard, after the Masovian Voivodship and Greater Poland Voivodeship which are the largest provinces in Poland (Statistics Poland, LDB, 2022). Therefore, presumably, the primary source of heavy metals in analyzed municipal sewage sludges is untreated wastewater produced by various industry branches.

### Heavy metal speciation

**Fig. 3:**
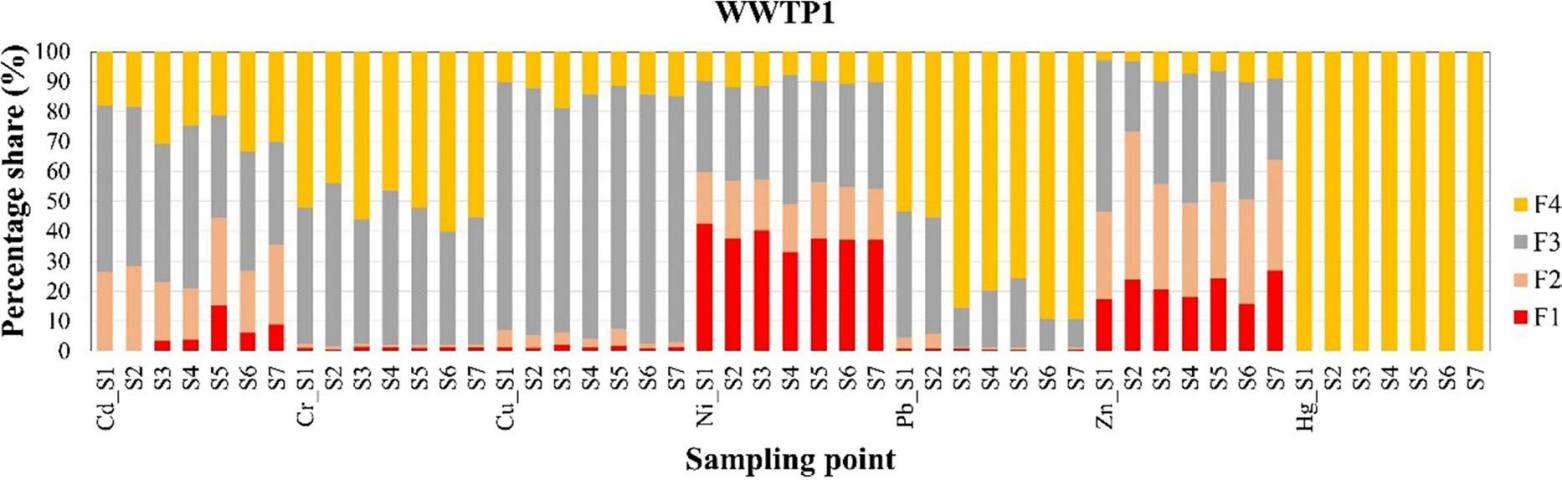
Distribution of heavy metals in different speciation fractions of sludge samples from WWTP1

**Fig. 4:**
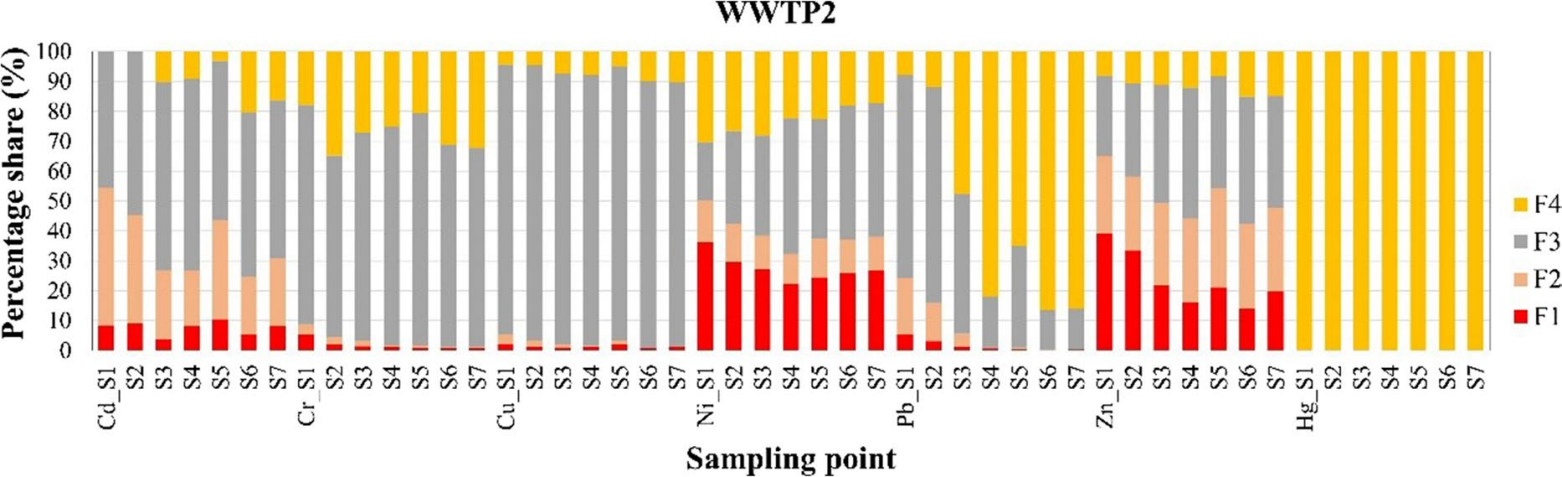
Distribution of heavy metals in different speciation fractions of sludge samples from WWTP2

The BCR sequential extraction assisted with ultrasound allows for the separation of four speciation fractions of heavy metals from sewage sludge (Tytła et al., 2022). The four fractions are as follows: acid soluble/exchangeable fraction (F1), a reducible fraction (F2), an oxidizable fraction (F3), and a residual fraction (F4) (optional one). These fractions provide insights into the forms, in which metals are bound within the sewage sludge. Fraction F1 is often associated with HMs bound to carbonates; F2 is typically associated with HMs bound to manganese (Mn) and iron (Fe) oxides; F3 comprises HMs that are bound to organic matter and sulfides, while F4 represents the fraction of HMs that are strongly bound to the solid matrix and are not easily extractable under the conditions of the sequential extraction procedure (Ure et al., 1993; Álvarez et al., 2002). Among them, the F1 fraction is characterized by the highest mobility and potential bioavailability, and it is of particular concern because it has the potential to directly impact the quality of water and soil, especially when sewage sludge is used for agricultural purposes (Nkinahamira et al., 2019; You et al., 2021). The second fraction is also mobile, but not as much as the first one, while F3 and F4 are both immobile. The obtained results were presented as the percentage of total concentrations of HMs in sludge samples (Figs. 3 and 4). Considering the share of individual HM in the most mobile fraction, in both cases, Zn and Ni had the most potential for migration, while Cd was to a lesser extent. The share of Zn and Ni in F1 accounted for 15.9 to 27.0% (WWTP1) and 14.0 to 39.1% (WWTP2), as well as from 33.2 to 42.6% (WWTP2) and 22.2 to 36.1% (WWTP2), respectively, whereas for Cd from 3.6 to 15.4% (WWTP1) and 3.7 to 10.5% (WWTP2). Comparable results were obtained by Li et al. (2015) who showed that the contribution of Zn, Ni, and Cd in the first fraction was approx. 40–50%; 40%, and 20%, respectively. The high potential for migration of Zn and Ni was also confirmed by other scientists (Gusiatin et al., 2018; Kowalik et al., 2021). In turn, considering the summarized percentage concentrations of Zn, Ni, and Cd in fractions F1 and F2, their share was in the range of 46.6 to 73.2% (Zn); 49.0 to 59.6% (Ni); 21.1 to 44.4% (Cd), and 42.3 to 65.2% (Zn); 32.3 to 50.2% (Ni); 24.6 to 54.4% (Cd), for WWTP1 and WWTP2, respectively. Based on the above-presented data, the real level of environmental risk for a given metal can be higher. Moreover, Cr and Cu were found to be principally distributed in fractions F3 and F4, regardless of at which point, in the processing line, the individual sludge sample was taken, while Hg was found only in residual fraction. This means that Cr, Cu, and Hg should not constitute a threat to the natural environment. Moreover, the recovery rates (R) for the HMs (excluding Hg) investigated in this study were in the range from 70 to 132% (WWTP1) and 95 to 133% (WWTP2).

In the discussed study, we noticed that SS at different stages of its treatment demonstrated higher contents of HMs that were predominantly bound to the immobile fractions. However, there were a few exceptions observed for Zn and Ni concentrations. It was also found that the main processes that can influence the binding of heavy metals and consequently the level of potential ecological risk that these contaminants pose to the natural environment are the activated sludge process (S3) and anaerobic digestion (S6). As it is commonly known, the activated sludge process is carried out in aerated biological reactors, where the dissolved oxygen concentration undergoes substantial fluctuations (Tytła et al., 2023). In the WWTP1 and WWTP2, the excess sludge samples (S3), which had previously undergone the activated sludge process, displayed an elevation in both pH and redox potential values. All these factors may result in a reduction of HM mobility. The reduction of heavy metals mobility resulted from the activated sludge process was noted in relation to Cd, Pb, and Zn (WWTP1) and to Cd, Cr, Cu, Ni, Pb, and Zn (WWTP2). Similar observations were also reported in previous research (Tytła, 2019). In turn, during anaerobic digestion, where the organic matter content undergoes reduction, metals bound to OM are released. This may increase the share of these elements in immobile fractions, especially in fraction F3 which was observed in previous work (Tytła et al., 2023). We observed the above-mentioned tendency only in relation to Cd, Cu, Ni, and Zn (WWTP1) and to Cd, Ni, and Zn (WWTP2). We found a similar tendency in previous study (Tytła et al., 2016). The reduction of the HMs mobility is a positive phenomenon concerning the environmental protection against its secondary pollution with toxic metals.

### Risk assessment

Assessment of the potential ecological and human health risks in the conducted study was applied to the dewatered sewage sludge (S7). This type of SS refers to the final product or waste generated after the various wastewater treatment processes have been completed. Therefore, the assessment of potential risks associated with dewatered sewage sludge is crucial to ensure that its disposal does not pose adverse effects on the natural environment or human health. In the absence of significant seasonal changes in the concentrations of HMs in the dewatered sludge samples, except for Zn, the average concentrations of HMs were used for the evaluation of potential risks.

### Potential ecological risk assessment

One of the key issues in the process of ecological risk assessment is the choice of the appropriate reference concentrations for the particular element. In the case of sewage sludge, the most reference background values are mainly the concentrations of HMs in the earth’s crust or the soil (Zhang et al., 2020; Yakamercan et al., 2021; Sundha et al., 2022). However, these values depend on the geological context, climate, and also other local factors and may differ for different metals. Therefore, it is crucial to consider various types of background values when conducting environmental assessments. In this study, to calculate the Geoaccumulation Index (*I*_*geo*_) and Potential Ecological Risk Factor (ER) values, we used the concentrations of HMs in the earth’s crust and the top soils of Europe (as background values), given by Kabata-Pendias (2011). The decision for using average values of HMs in top soils of Europe was made due to the lack of specific guidelines for soils in the area under consideration. Table 7 presents the results of the ecological risk assessment conducted for the HMs in the dewatered sludge samples.

**Table 7:** Ecological risk of the heavy metals in the dewatered sludge from two WWTPs

WWTP	Index	Cd	Cr	Cu	Ni	Pb	Zn	Hg
WWTP1	*I_geo_	5.0 (H-EC)	− 0.9 (PUC)	1.5 (MC)	2.2 (M-HC)	1.7 (MC)	3.4 (HC)	1.4 (MC)
	**I_geo_	3.5 (HC)	− 0.9 (PUC)	3.2 (HC)	1.3 (MC)	0.6 (U-MC)	3.4 (HC)	1.6 (MC)
	*ER	1427.9 (VHR)	1.6 (LR)	21.8 (LR)	34.1 (LR)	23.9 (LR)	15.9 (LR)	160.1 (HR)
	**ER	510.0 (VHR)	1.7 (LR)	69.4 (MR)	18.4 (LR)	11.2 (LR)	16.3 (LR)	183.7 (HR)
	RAC;%	8.9 (LR)	1.2 (LR)	1.4 (LR)	37.2 (HR)	0.6 (NR)	27.0 (MR)	0.0 (NR)
WWTP2	*I_geo_	6.9 (EC)	0.8 (U-MC)	1.6 (MC)	0.7 (U-MC)	3.7 (HC)	4.3 (H-EC)	− 0.2 (PUC)
	**I_geo_	5.4 (EC)	0.9 (U-MC)	3.3 (HC)	− 0.2 (PUC)	2.6 (M-HC)	4.3 (H-EC)	− 0.05 (PUC)
	*ER	5203.0 (VHR)	5.3 (LR)	22.7 (LR)	12.4 (LR)	96.8 (CR)	29.5 (LR)	50.7 (MR)
	**ER	1858.2 (VHR)	5.6 (LR)	72.1 (MR)	6.7 (LR)	45.4 (MR)	30.3 (LR)	58.1 (MR)
	RAC;%	8.1 (LR)	0.8 (NR)	1.2 (LR)	26.8 (MR)	0.3 (NR)	19.8 (MR)	0.0 (NR)

*In reference to the Earth’s crust; ** in reference to the top soils of Europe (Kabata-Pendias, 2011)

The values of *I*_*geo*_ showed that dewatered sludge samples are more or less contaminated (enriched) in most of the analyzed heavy metals, which may also prove their anthropogenic origin. The *I*_*geo*_ values of HMs in SS samples, ranked from highest to lowest, were found to be Cd > Zn > Ni > Pb > Cu > Hg > Cr and Cd > Zn > Cu > Hg > Ni > Pb > Cr (WWTP1), as well as Cd > Zn > Pb > Cu > Cr > Ni > Hg and Cd > Zn > Cu > Pb > Cr > Hg > Ni (WWTP2). In the first case, the values of *I*_*geo*_ were calculated taking into account the concentrations of HMs in the earth’s crust, while in the second one, in the top soils of Europe. Despite the differences between the concentrations of individual HMs in two different reference backgrounds, the sludge samples were high to extremely and extremely contaminated with Cd and Zn, respectively, and to a lesser extent with Cu and Pb. The above-presented results are similar to those achieved by other researchers. For example, Li et al. (2015) indicate that sewage sludge from several WWTPs was highly contaminated with Cd (*I*_*geo*_ = 3.3.) and moderately to highly contaminated with Zn (*I*_*geo*_ = 2.7). Also, other scientists confirmed that cadmium can be the main pollutant in SS and indicate that the *I*_*geo*_ values of analyzed elements were in the decreasing order: Cd > Pb > Hg > Cr > As (Zhang et al. 2020). Similar results were presented by Sundha et al. (2022), who showed that sludge samples were high to extremely contaminated with cadmium (*I*_*geo*_ = 4.1 to 4.12) and moderately to highly and highly to extremely polluted with zinc (*I*_*geo*_ = 2.4 to 4.7). In contrary to our research, Kowalik et al. (2022) indicated that SS from four WWTPs in Poland (Świętokrzyskie Voivodeship) were heavily to extremely and extremely contaminated with Zn, and heavily to heavily to extremely with Cd.

The ER values for the analyzed elements in the dewatered sludges were in the order as follows: Cd > Hg > Ni > Pb > Cu > Zn > Cr and Cd > Hg > Cu > Ni > Zn > Pb > Cr (WWTP1) and Cd > Pb > Hg > Zn > Cu > Ni > Cr and Cd > Cu > Hg > Pb > Zn > Ni > Cr (WWTP2). The obtained results refer to the concentration levels of metals in the Earth’s crust and in the top soils of Europe, respectively. It was found that Cd posed a very high environmental risk (WWTP1 and WWTP2), Hg posed a moderate (WWTP2) to high risk (WWTP1), and Pb a considerable risk (WWTP2), whereas other HMs presented a moderate or low risk. The high ER values with cadmium and mercury are most probably associated with the high values of the toxic response factor, which are 30 and 40, for Cd and Hg, respectively, whereas for the rest of the analyzed metals from 1 to 5. The observed tendency (levels of risk) was similar, despite some differences in the values of HMs (especially for Cd and Cu) in the earth’s crust and in the top soils of Europe. The obtained results are consistent with those presented by other scientists who collected sludge samples from the three WWTPs, located at different sites in urban areas and found that Cd (ER = 662.9) and Hg (ER = 529.7) posed a very high risk (Li et al., 2015). Similar results were presented by researchers from China (Zhang et al., 2020), who indicated that ER values of elements analyzed in sludge samples from eight WWTPs, were found in the following order Cd > Hg > As > Pb > Cr. They also indicate that among the discussed elements, only Cd posed a serious ecological risk. Therefore, it can be assumed that toxic HMs can pose a serious ecological risk, even when present in low concentrations. Moreover, Espinoza-Guillen et al. (2022) who examined sludge samples from several WWTPs located in Peru indicated that Pb poses a low (ER = 19.6) to high (ER = 80.8) ecological risk. Thus, it can be concluded that the outcomes described in the abovementioned works are well corresponding with the results obtained in our study.

According to the values of the Risk Assessment Code (RAC) index, the mean percentages of HMs in the dewatered sludges associated with the fraction F1 ranked in the decreasing order: Ni > Zn > Cd > Cu > Cr > Pb > Hg, for both WWTPs. It was found that Ni posed medium (RAC = 26.7%; WWTP2) to high risk (RAC = 37.2%; WWTP1) while Zn medium risk (RAC = 27.0% for WWTP1; RAC = 19.8% for WWTP2). Other metals posed low risk (Cd, Cr, and Cu) or did not pose an ecological risk at all (Pb and Hg). Similar results to those obtained in this study were described by researchers from China (You et al., 2021), who collected SS samples from WWTPs situated in different locations of Huainan City (an urban area). They ranked the average values of RAC for the analyzed HMs in the following order: As > Ni(17.4%) > Zn(14.4%) > Pb > Cd > Cu > Cr, where Ni and Zn were in second and third place, right after As. The dominant share of Zn (RAC = 63.5%) in the most mobile fraction was also indicated by Kowalik et al. (2021) who studied sludge samples from a couple of WWTPs, located in the southern part of central Poland.

### Human health risk assessment

#### Exposure assessment

The mean values of the average daily exposure dose (ADD) of HMs in SS samples are shown in Table 8. The obtained results indicate that the dominant pathway, through which HMs can enter the human body, was ingestion, for both demographical groups (adults and children). The above findings well correspond with the results presented by other scientists (Duan et al., 2017; Duan & Feng, 2021; Espinoza-Guillen et al., 2022; Yakamercan & Aygün, 2023). The highest values of *ADD*_*ing*_, *ADD*_*inh*_, and *ADD*_*dermal*_ to non-carcinogenic HMs for adults and children were indicated to Zn (WWTP1 and WWTP2) while for carcinogenic ones to Ni and Cr (WWTP1) and to Pb and Cr (WWTP2). Moreover, the mean values of average daily doses (ADDs) taken through accidental ingestion, inhalation, and dermal contact, posed by all non-carcinogenic HMs for adults were 6.19 × 10^−4^ mg·kg^−1^·day^−1^ (WWTP1) and 1.06 × 10^−3^ mg·kg^−1^·day^−1^ (WWTP2) while for children 5.41 × 10^−3^ mg·kg^−1^·day^−1^ (WWTP1) and 9.27 × 10^−3^ mg·kg^−1^·day^−1^ (WWTP2), respectively. In turn, mean values of ADDs posed by all carcinogenic HMs for adults were 4.30 × 10^−5^ mg·kg^−1^·day^−1^ (WWTP1) and 9.14 × 10^−5^ mg·kg^−1^·day^−1^ (WWTP2) whereas for children 7.52 × 10^−5^ mg·kg^−1^·day^−1^ (WWTP1) and 1.60 × 10^−4^ mg·kg^−1^·day^−1^ (WWTP2). The obtained results indicating that the magnitude of exposure rates for children was greater than for adults suggest that children may be more vulnerable to the health risks associated with exposure to HMs in sewage sludge. A similar tendency was also noted by other researchers (Duan et al., 2017; Yakamercan et al., 2021; Espinoza-Guillen et al., 2022).

**Table 8:** The mean values of average daily exposure doses (ADDs) of heavy metals in the dewatered sludges from two WWTPs

WWTP	Life range	Index	Cd	Cr	Cu	Ni	Pb	Zn	Hg
			mg·kg^−1^·day^−1^						
WWTP1	Adults	*ADD*_*ing*_	2.79 × 10^-6^	4.63 × 10^-5^	3.29 × 10^-4^	8.01 × 10^-5^	4.22 × 10^-5^	1.52 × 10^-3^	3.84 × 10^-7^
		*ADD*_*inh*_	4.11 × 10^-10^	6.81 × 10^-9^	4.84 × 10^-8^	1.18 × 10^-8^	6.20 × 10^-9^	2.24 × 10^-7^	5.64 × 10^-11^
		*ADD*_*dermal*_	1.11 × 10^-8^	1.85 × 10^-7^	1.31 × 10^-6^	3.20 × 10^-7^	1.68 × 10^-7^	6.06 × 10^-6^	1.53 × 10^-9^
		*ADD*	2.81 × 10^-6^	4.65 × 10^-5^	3.30 × 10^-4^	8.04 × 10^-5^	4.23 × 10^-5^	1.53 × 10^-3^	3.85 × 10^-7^
	Children	*ADD*_*ing*_	4.89 × 10^-6^	8.10 × 10^-5^	2.88 × 10^-3^	1.40 × 10^-4^	7.38 × 10^-5^	1.33 × 10^-2^	3.36 × 10^-6^
		*ADD*_*inh*_	1.37 × 10^-10^	2.26 × 10^-9^	8.04 × 10^-8^	3.92 × 10^-9^	2.06 × 10^-9^	3.72 × 10^-7^	9.38 × 10^-11^
		*ADD*_*dermal*_	1.37 × 10^-8^	2.27 × 10^-7^	8.06 × 10^-6^	3.93 × 10^-7^	2.07 × 10^-7^	3.72 × 10^-5^	9.40 × 10^-9^
		*ADD*	4.90 × 10^-6^	8.13 × 10^-5^	2.89 × 10^-3^	1.41 × 10^-4^	7.40 × 10^-5^	1.33 × 10^-2^	3.37 × 10^-6^
WWTP2	Adults	*ADD*_*ing*_	1.02 × 10^-5^	1.55 × 10^-4^	3.42 × 10^-4^	2.90 × 10^-5^	1.70 × 10^-4^	2.83 × 10^-3^	1.21 × 10^-7^
		*ADD*_*inh*_	1.50 × 10^-9^	2.27 × 10^-8^	5.03 × 10^-8^	4.27 × 10^-9^	2.51 × 10^-8^	4.16 × 10^-7^	1.79 × 10^-11^
		*ADD*_*dermal*_	4.06 × 10^-8^	6.17 × 10^-7^	1.36 × 10^-6^	1.16 × 10^-7^	6.80 × 10^-7^	1.13 × 10^-5^	4.84 × 10^-10^
		*ADD*	1.02 × 10^-5^	1.55 × 10^-4^	3.43 × 10^-4^	2.92 × 10^-5^	1.71 × 10^-4^	2.84 × 10^-3^	1.22 × 10^-7^
	Children	*ADD*_*ing*_	1.78 × 10^-5^	2.71 × 10^-4^	2.99 × 10^-3^	5.08 × 10^-5^	2.98 × 10^-4^	2.47 × 10^-2^	1.06 × 10^-6^
		*ADD*_*inh*_	4.98 × 10^-10^	7.56 × 10^-9^	8.36 × 10^-8^	1.42 × 10^-9^	8.33 × 10^-9^	6.91 × 10^-7^	2.97 × 10^-11^
		*ADD*_*dermal*_	4.99 × 10^-8^	7.58 × 10^-7^	8.37 × 10^-6^	1.42 × 10^-7^	8.35 × 10^-7^	6.92 × 10^-5^	2.97 × 10^-9^
		*ADD*	1.79 × 10^-5^	2.71 × 10^-4^	3.00 × 10^-3^	5.10 × 10^-5^	2.99 × 10^-4^	2.48 × 10^-2^	1.07 × 10^-6^

#### Non-carcinogenic and carcinogenic risk assessment

Potential human health risks for non-carcinogenic and carcinogenic heavy metals present in the analyzed sewage sludge samples are shown in Table 9. For the assessment of non-carcinogenic risk for adults and children exposed to metals contained in sludge samples, the values of hazard quotient (HQ) and hazard index (HI) were used. The obtained results indicated that the mean values of HQs for non-carcinogenic HMs followed the order: ingestion > dermal contact > inhalation. The presented tendency is consistent with the literature data (Agoro et al., 2020; Yakamercan et al., 2021). The highest values for *HQ*_*ing*_, *HQ*_*dermal*_, and *HQ*_*inh*_ were caused by Cu (WWTP1) and Zn (WWTP2), both for adults and children. The above findings were confirmed by the values of HI. Moreover, also other scientists confirmed that Cu was the most prevailing component for HI (Duan et al., 2017; Nyashanu et al., 2023). However, in any case, the values of HI and HQ for analyzed HMs did not exceed the threshold value (1). Therefore, it can be stated that in the case of the dewatered sludges from the discussed WWTPs, there is no potential human health risk associated with the presence of non-carcinogenic HMs (Cu, Zn, and Hg), if the sludge samples were used for agricultural purposes.

**Table 9:** Human health risks from heavy metals in the dewatered sludges from two WWTPs

WWTP	Life range	HMs	HQ_ing_	HQ_inh_	HQ_dermal_	HI	HMs	CR_ing_	CR_inh_	CR_dermal_	TCR
WWTP1	Adults	Cu	8.22 × 10^-3^	1.20 × 10^-6^	1.09 × 10^-4^	8.33 × 10^-3^	Cd	1.76 × 10^-5^	2.59 × 10^-9^	-	1.76 × 10^-5^
		Zn	5.07 × 10^-3^	7.45 × 10^-7^	1.01 × 10^-4^	5.17 × 10^-3^	Cr	2.32 × 10^-5^	2.86 × 10^-7^	3.70 × 10^-6^	2.71 × 10^-5^
		Hg	1.28 × 10^-3^	1.88 × 10^-7^	8.32 × 10^-7^	1.28 × 10^-3^	Ni	1.36 × 10^-4^	9.90 × 10^-9^	1.36 × 10^-5^	1.50 × 10^-4^
		-	-	-	-	-	Pb	3.58 × 10^-7^	2.60 × 10^-10^	1.43 × 10^-6^	1.79 × 10^-6^
	Children	Cu	7.20 × 10^-2^	2.00 × 10^-6^	6.72 × 10^-4^	7.26 × 10^-2^	Cd	3.08 × 10^-5^	8.61 × 10^-10^	-	3.08 × 10^-5^
		Zn	4.43 × 10^-2^	1.24 × 10^-6^	6.21 × 10^-4^	4.50 × 10^-2^	Cr	4.05 × 10^-5^	9.51 × 10^-8^	4.54 × 10^-6^	4.52 × 10^-5^
		Hg	1.12 × 10^-2^	3.13 × 10^-7^	5.11 × 10^-6^	1.12 × 10^-2^	Ni	2.38 × 10^-4^	3.29 × 10^-9^	1.67 × 10^-5^	2.55 × 10^-4^
		-	-	-	-	-	Pb	6.27 × 10^-7^	8.66 × 10^-11^	1.76 × 10^-6^	2.38 × 10^-6^
WWTP2	Adults	Cu	8.55 × 10^-3^	1.25 × 10^-6^	1.14 × 10^-4^	8.66 × 10^-3^	Cd	6.41 × 10^-5^	9.43 × 10^-9^	-	6.42 × 10^-5^
		Zn	9.42 × 10^-3^	1.39 × 10^-6^	1.88 × 10^-4^	9.61 × 10^-3^	Cr	7.73 × 10^-5^	9.55 × 10^-7^	1.23 × 10^-5^	9.06 × 10^-5^
		Hg	4.05 × 10^-4^	5.95 × 10^-8^	2.63 × 10^-7^	4.05 × 10^-4^	Ni	4.94 × 10^-5^	3.59 × 10^-9^	4.92 × 10^-6^	5.43 × 10^-5^
		-	-	-	-	-	Pb	1.45 × 10^-6^	1.05 × 10^-9^	5.78 × 10^-6^	7.23 × 10^-6^
	Children	Cu	7.48 × 10^-2^	2.08 × 10^-6^	6.98 × 10^-4^	7.55 × 10^-2^	Cd	1.12 × 10^-4^	3.14 × 10^-9^	-	1.12 × 10^-4^
		Zn	8.24 × 10^-2^	2.30 × 10^-6^	1.15 × 10^-3^	8.36 × 10^-2^	Cr	1.35 × 10^-4^	3.17 × 10^-7^	1.52 × 10^-5^	1.51 × 10^-4^
		Hg	3.54 × 10^-3^	9.90 × 10^-8^	1.62 × 10^-6^	3.54 × 10^-3^	Ni	8.64 × 10^-5^	1.19 × 10^-9^	6.05 × 10^-6^	9.25 × 10^-5^
		-	-	-	-	-	Pb	2.54 × 10^-6^	3.50 × 10^-10^	7.10 × 10^-6^	9.63 × 10^-6^

To assess the carcinogenic risk for both demographic groups exposed to HMs in the sludge samples, the values of carcinogenic risk (CR) and total carcinogenic risk (TCR) were applied. The conducted calculations revealed that for considered WWTPs, the mean values of CRs for carcinogenic HMs decreased in the following order: ingestion > dermal contact > inhalation. The highest values for *CR*_*ing*_ and *CR*_*dermal*_ for adults were indicated to Ni (WWTP1) and Cr (WWTP2), while for *CR*_*inh*_ to Cr (WWTP1 and WWTP2), in turn, for children to Ni (WWTP1) and Cr/Cd (WWTP2); Ni (WWTP1) and Cr (WWTP2) and Cr (WWTP1 and WWTP2), respectively. The values of *CR*_*ing*_ indicate that there is a carcinogenic risk of exposure to Ni in the dewatered SS from WWTP1, both for adults and children, while in the case of sludge samples from WWTP2 regarding Cr and Cd, but only for children. In both cases, the values of *CR*_*ing*_ are higher than 1 × 10^−4^. Moreover, also the values of TCR confirmed that in the case of Ni (WWTP1), there is a carcinogenic risk, both for adults (1.50 × 10^−4^) and children (2.55 × 10^−4^), while in the case of Cr and Cd (WWTP2), only for children, i.e., 1.51 × 10^−4^ and 1.12 × 10^−4^, respectively. This means, that the agricultural use of the analyzed sludges may cause carcinogenic risk to both demographic groups; however, higher risk occurs for children. Similar observations were made by other researchers (Duan & Feng, 2021; Espinoza-Guillen et al., 2022; Qayoom et al., 2022). The conducted studies also indicate that for Cd, Cr, and Pb (adults and children; WWTP1); Cd, Cr, Ni, and Pb (adults; WWTP2); and Ni and Pb (children; WWTP2), the TCRs are acceptable (1 × 10^−4^–1 × 10^−6^). Therefore, both for the HMs that pose carcinogenic risk, and for those elements that pose acceptable risk, it is recommended to take appropriate steps to reduce the concentrations of HMs in sewage sludge intended for agricultural purposes, to minimize the level of potential human health risk. As it is commonly known, the processes used in WWTPs do not guarantee that heavy metals will be removed from sewage sludge. However, there are groups of techniques, which may reduce the concentrations of these elements, for example, the chemical (acidification, alkalization, and ion exchange treatment), physical (heat treatment and electroremediation), and biological (vermicomposting, application of biosurfactant, and bioleaching) ones. Unfortunately, methods which belong to the first two groups are expensive and may pose a threat to the natural environment, while biological techniques are promising but also time-consuming, and still were not tested on a full scale (Camargo et al., 2016).

## Conclusions

This study represents a comprehensive assessment of both ecological and human health risks posed by heavy metals in sewage sludge from municipal WWTPs situated in Silesian Voivodeship, the most industrialized region in Poland. The conducted research showed that Zn, Cu, and Pb are present in the highest concentrations in various types of sludges, while Cd and Hg are in the lowest. Moreover, positive significant correlations of most of the examined elements were found which means that they may have the same source of origin. Taking into account the characteristic of the study area, it is highly probable that the main source of these metals in sewage sludge is wastewater from the industry. Despite the fact that dewatered sludge samples analyzed in this study meet standards for their use for agricultural purposes, the values of *I*_*geo*_ revealed that they are mainly contaminated with Cd, Zn, and to a lesser extent with Cu and Pb. This means that the presence of these elements in sewage sludge may result in soil contamination and even secondary environmental pollution. Assessment of the ecological risk, based on the values of the ER index, which also includes the toxicity of elements, revealed that the most serious risk is being posed by Cd and Hg, which are present in the lowest concentrations. On the contrary, based on the RAC values and the chemical speciation forms in which heavy metals are present, it was found that Ni and Zn pose a significant ecological risk. As it was mentioned, the occurrence of HMs in sewage sludge is often attributed to anthropogenic activities, particularly those related to chemical or heavy industries. The obtained results indicate that the total content of heavy metals and their speciation forms in the sewage sludge are inseparable elements of the ecological risk assessment. Moreover, for a precise interpretation of the obtained results, it is crucial to consider various types of background values. The results of non-carcinogenic and carcinogenic risks analysis revealed that the dominant route of HMs exposure was ingestion. It was revealed that for both WWTPs, there is no potential human health risk associated with the presence of non-carcinogenic HMs (Cu, Zn, and Hg) in the analyzed sludge samples. However, it was indicated that in the case of Ni (WWTP1), there is a carcinogenic risk, both for adults and children, while in the case of Cr and Cd (WWTP2), only for children.

In summary, the obtained results proved that municipal sewage sludge produced in Poland may pose potential risks. Therefore, to avoid secondary pollution of the natural environment and harmful human health effects, decisive action to reduce the concentrations of heavy metals in sewage sludge must be taken. It should also be stated that the assessment of the suitability of sewage sludge for agricultural purposes should be supplemented with a comprehensive risk analysis.

## Data Availability

The datasets generated during the current study are available from the corresponding author on reasonable request.

## References

[CR1] Agoro MA, Adeniji AO, Adefisoye MA, Okoh OO (2020). Heavy metals in wastewater and sewage sludge from selected municipal treatment plants in Eastern Cape Province, South Africa. Water.

[CR2] Álvarez EA, Callejón Mochón M, Jiménez Sánchez JC, Ternero Rodríguez M (2002). Heavy metal extractable forms in sludge from wastewater treatment plants. Chemosphere.

[CR3] Baran A, Tarnawski M, Koniarz T (2016). Spatial distribution of trace elements and ecotoxicity of bottom sediments in Rybnik reservoir, Silesian–Poland. Environmental Science and Pollution Research.

[CR4] Bochenek D, Dawgiałło U, Gorzkowska E, Hejne J, Kiełczykowska A, Kruszewska D, Nieszała A, Nowakowska B, Sulik J, Wichniewicz A, Wrzosek A, Supervised by Domańska W. (2022). *Environment 2022* (Statistical analyses).

[CR5] Camargo FP, Tonello PS, dos Sanots ACA, Duarte ICS (2016). Removal of toxic metals from sewage sludge through chemical, physical, and biological treatments - a review. Water, Air, & Soil Pollution.

[CR6] Cieślik BM, Namieśnik J, Konieczka P (2015). Review of sewage sludge management: standards, regulations and analytical methods. Journal of Cleaner Production.

[CR7] Council Directive of 12th June 1986 on the protection of the environment, and in particular of the soil, when sewage sludge is used in agriculture (Directive 86/278/EEC). Retrieved March 23, 2023, from: https://eur-lex.europa.eu/legal-content/PL/TXT/PDF/?uri=CELEX:31986L0278&from=EN.

[CR8] Duan B, Zhang W, Zheng H, Wu C, Zhang Q, Bu Y (2017). Comparison of health risk assessments of heavy metals and As in sewage sludge from wastewater treatment plants (WWTPs) for adults and children in the urban district of Taiyuan, China. International Journal of Environmental Research and Public Health.

[CR9] Duan B, Feng Q (2021). Comparison of the potential ecological and human health risks of heavy metals from sewage sludge and livestock manure for agricultural use. Toxics.

[CR10] Duan B, Feng Q (2022). Risk assessment and potential analysis of the agricultural use of sewage sludge in central Shanxi Province. International Journal of Environmental Research and Public Health.

[CR11] Espinoza-Guillen JA, Alderete-Malpartida MB, Gallegos-Huamán RL, Paz-Rosales YM, Domínguez-Vivar RM, Bujaico-León C (2022). Ecological risk assessment and identification of sources of heavy metals contamination in sewage sludge from municipal wastewater treatment plants in the Metropolitan Area of Lima-Callao.

[CR12] Eurostat Data Base (2019) https://ec.europa.eu/eurostat/

[CR13] Eurostat Data Base (2020) https://ec.europa.eu/eurostat/

[CR14] Eurostat Data Base (2021) https://ec.europa.eu/eurostat/

[CR15] Geng H, Xu Y, Zheng L, Gong H, Dai L, Dai X (2020). An overview of removing heavy metals from sewage sludge: achievements and perspectives. Environmental Pollution.

[CR16] Google Maps (Map of Europe) (n.d.). Retrieved June 15, 2023, from: https://www.google.pl/maps/

[CR17] Gusiatin ZM, Kulikowska D, Klik BK, Hajdukiewicz K (2018). Ecological risk assessment of sewage sludge from municipal wastewater treatment plants: a case study. Journal of Environmental Science and Health, Part A.

[CR18] Gwebu S, Tavengwa NT, Klink MJ, Mtunzi FM, Modise SJ, Pakade VE (2017). Quantification of Cd, Cu, Pb and Zn from sewage sludge by modified-BCR and ultrasound assisted-modified BCR sequential extraction methods. African Journal of Pure and Applied Chemistry.

[CR19] Håkanson L (1980). An ecological risk index for aquatic pollution control. A sedimentological approach. Water Research.

[CR20] International Agency for Research on Cancer (IARC) (2012). *IARC monographs on the evaluation of carcinogenic risks to humans. Arsenic, metals, fibers and dusts. A review of human carcinogens (No. 100C)*.

[CR21] Kabata-Pendias A (2011). *Trace elements in soils and plants*.

[CR22] Kendir E, Kentel E, Sanin FD (2015). Evaluation of heavy metals and associated health risks in a metropolitan wastewater treatment plant’s sludge for its land application. Human and Ecological Risk Assessment: An International Journal.

[CR23] Kowalik R, Latosińska J, Gawdzik J (2021). Risk analysis of heavy metal accumulation from sewage sludge of selected wastewater treatment plants in Poland. Water.

[CR24] Kowalik R, Gawdzik J, Bąk-Patyna P, Ramiączek P, Jurišević N (2022). Risk analysis of heavy metals migration from sewage sludge of wastewater treatment plants. International Journal of Environmental Research and Public Health.

[CR25] Li J, Luo G, Gao J, Yuan S, Du J, Wang Z (2015). Quantitative evaluation of potential ecological risk of heavy metals in sewage sludge from three wastewater treatment plants in the main urban area of Wuxi, China. Chemistry and Ecology.

[CR26] Li J, Luo G, Xu J (2019). Fate and ecological risk assessment of nutrients and metals in sewage sludge from ten wastewater treatment plants in Wuxi City, China. Bulletin of Environmental Contamination and Toxicology.

[CR27] Miletić A, Lučić M, Onjia A (2023). Exposure factors in health risk assessment of heavy metal(loid)s in soil and sediment. Metals.

[CR28] Milik J, Pasela R, Lachowicz M, Chalamonski M (2017). The concentration of trace elements in sewage sludge from wastewater treatment plant in Gniewino. *Journal of*. Ecological Engineering.

[CR29] Müller G (1969). Index of geoaccumulation in sediments of the Rhine River. GeoJournal.

[CR30] Nkinahamira F, Suanon F, Chi Q, Li Y, Feng M, Huang X, Yu CP, Sun Q (2019). Occurrence, geochemical fractionation, and environmental risk assessment of major and trace elements in sewage sludge. Journal of Environmental Management.

[CR31] Nyashanu PN, Shafodino FS, Mwapagha LM (2023). Determining the potential human health risks posed by heavy metals present in municipal sewage sludge from a wastewater treatment plant. Scientific African.

[CR32] Perin G, Craboledda L, Lucchese M, Cirillo R, Dotta L, Zanetta ML, Oro AA, Lakkas TD (1985). Heavy metal speciation in the sediments of northern Adriatic Sea. A new approach for environmental toxicity determination. *Heavy Metals in the Environment*.

[CR33] Polish Committee for Standardization (Polski Komitet Normalizacyjny – PKN) (2004). *Characteristics of sewage sludge – determination of dry residue and water content*.

[CR34] Polish Committee for Standardization (Polski Komitet Normalizacyjny – PKN) (2004). *Characteristics of sewage sludge – determination of loss on ignition of dry matter*.

[CR35] Polish Committee for Standardization (Polski Komitet Normalizacyjny – PKN) (2004). *Characterization of waste – determination of total organic carbon (TOC) in waste, sludges and sediments*.

[CR36] Qayoom U, Bhat SU, Ahmad I, Kumar A (2022). Assessment of potential risks of heavy metals from wastewater treatment plants of Srinagar city, Kashmir. International Journal of Environmental Science and Technology.

[CR37] Regulation of the Minister of Environment of 6th February 2015 on the Municipal Sewage Sludge (J. L. 2015, Item 257). Retrieved March 23, 2023, from: https://isap.sejm.gov.pl/isap.nsf/DocDetails.xsp?id=wdu20150000257

[CR38] Risk Assessment Information System (RAIS). Chemical toxicity. Chemical Data Profiles 2023. Retrieved September 3, 2023, from: https://rais.ornl.gov/

[CR39] Statistics Poland, Local Data Bank LDB (2022) https://bdl.stat.gov.pl/

[CR40] Sundha, P., Basak, N., Rai, A. K., Chandra, P., Bedwal, S., Yadav, G., Yadav, R. K., & Sharma, P. C. (2022). Characterization and ecotoxicological risk assessment of sewage sludge from industrial and non-industrial cities. *Environmental Science and Pollution Research*. 10.1007/s11356-022-21648-2 10.1007/s11356-022-21648-2 35779215

[CR41] Tessier A, Campbell PGC, Bisson M (1979). Sequential extraction procedure for the speciation of particulate trace metals. Analytical Chemistry.

[CR42] Tytła M, Widziewicz K, Zielewicz Z (2016). Heavy metals and its chemical speciation in sewage sludge at different stages of processing. Environmental Technology.

[CR43] Tytła M (2019). Assessment of heavy metal pollution and potential ecological risk in sewage sludge from municipal wastewater treatment plant located in the most industrialized region in Poland—case study. International Journal of Environmental Research and Public Health.

[CR44] Tytła M, Widziewicz-Rzońca K, Bernaś Z (2022). A comparison of conventional and ultrasound-assisted BCR sequential extraction methods for the fractionation of heavy metals in sewage sludge of different characteristics. Molecules.

[CR45] Tytła M, Widziewicz-Rzońca K, Kernert J, Bernaś Z, Słaby K (2023). First Comprehensive analysis of potential ecological risk and factors influencing heavy metals binding in sewage sludge from WWTPs using the ultrasonic disintegration process. Water.

[CR46] United States Environmental Protection Agency (USEPA) (1989). *Risk assessment guidance for superfund, volume I, human health evaluation manual (part A)*.

[CR47] United States Environmental Protection Agency (USEPA) (1991). *Assessment guidance for superfund. Vol. I: human health evaluation manual*. Supplemental Guidance “Standard Default Exposure Factors”.

[CR48] United States Environmental Protection Agency (USEPA) (1995). *A guide to the biosolids risk assessments for the EPA part 503 part*.

[CR49] United States Environmental Protection Agency (USEPA) (2001). *Risk assessment guidance for superfund: vol III - part A, process for conducting probabilistic risk assessment*.

[CR50] United States Environmental Protection Agency (USEPA) (2002). *Supplemental guidance for developing soil screening levels for superfund sites*.

[CR51] Ure AM, Quevauviller P, Mantau H, Griepink B (1993). Speciation of heavy metals in soils and sediments. An account of the improvement and harmonization of extraction techniques undertaken under the auspices of the BCR of the Commission of the European Communities. International Journal of Environmental Analytical Chemistry.

[CR52] Wang C, Li XC, Ma HT, Qian J, Zhai JB (2006). Distribution of extractable fractions of heavy metals in sludge during the wastewater treatment process. Journal of Hazardous Materials.

[CR53] Xuan C, Jianfeng Z, Changshun S (2023). Characteristics and risk assessment of sewage sludge from urban wastewater treatment plants in Shaanxi Province, China. Environmental Monitoring and Assessment.

[CR54] Yakamercan E, Ari A, Aygün A (2021). Land application of municipal sewage sludge: human health risk assessment of heavy metals. Journal of Cleaner Production.

[CR55] Yakamercan E, Aygün A (2023). Health risk assessment of metal(loid)s for land application of domestic sewage sludge in city of Bursa, Türkiye. Environmental Monitoring and Assessment.

[CR56] You M, Hu Y, Yan Y, Yao J (2021). Speciation characteristics and ecological risk assessment of heavy metals in municipal sludge of Huainan, China. Molecules.

[CR57] Zhang H, Huang Y, Zhou S, Wei L, Guo Z, Li J (2020). Pollution level and risk assessment of heavy metals in sewage sludge from eight wastewater treatment plants in Wuhu City, China. Spanish Journal of Agricultural Research.

